# Landscape effects on demersal fish revealed by field observations and predictive seabed modelling

**DOI:** 10.1371/journal.pone.0189011

**Published:** 2017-12-11

**Authors:** Sophie A. M. Elliott, Alessandro D. Sabatino, Michael R. Heath, William R. Turrell, David M. Bailey

**Affiliations:** 1 Institute of Biodiversity, Animal Health and Comparative Medicine, University of Glasgow, Glasgow, United Kingdom; 2 Department of Mathematics and Statistics, University of Strathclyde, Glasgow, United Kingdom; 3 Marine Scotland Science, Marine Laboratory, Aberdeen, United Kingdom; University Zürich, SWITZERLAND

## Abstract

Nature conservation and fisheries management often focus on particular seabed features that are considered vulnerable or important to commercial species. As a result, individual seabed types are protected in isolation, without any understanding of what effect the mixture of seabed types within the landscape has on ecosystem functions. Here we undertook predictive seabed modelling within a coastal marine protected area using observations from underwater stereo-video camera deployments and environmental information (depth, wave fetch, maximum tidal speeds, distance from coast and underlying geology). The effect of the predicted substratum type, extent and heterogeneity or the diversity of substrata, within a radius of 1500 m around each camera deployment of juvenile gadoid relative abundance was analysed. The predicted substratum model performed well with wave fetch and depth being the most influential predictor variables. *Gadus morhua* (Atlantic cod) were associated with relatively more rugose substrata (Algal-gravel-pebble and seagrass) and heterogeneous landscapes, than *Melanogrammus aeglefinus* (haddock) or *Merlangius merlangus* (whiting) (sand and mud). An increase in *M*. *merlangus* relative abundance was observed with increasing substratum extent. These results reveal that landscape effects should be considered when protecting the seabed for fish and not just individual seabed types. The landscape approach used in this study therefore has important implications for marine protected area, fisheries management and monitoring advice concerning demersal fish populations.

## Introduction

Protecting species requires good knowledge of species distribution and the role of their habitat including the wider landscape effects. Unfortunately, there is often little information on landscape effects on fish abundance and survival [1–3]. This is particularly important given the mobility of many demersal and commercial fish species. It is well recognised that particular seabed types and the biodiversity they provide is of significance to demersal fish (e.g. [4,5]). The roles of different seabed types are especially important during demersal fish juvenile stages, where they are more vulnerable to predation (e.g.[3,6,7]). Understanding landscape ecology provides a more complete understanding of the ecological function and broader spatial pattern of fish [8]. Here, we define landscape as the composition, distribution, and topography of substratum types within a given area [7].

Fisheries affect fish populations through direct mortality as catch or bycatch and also through indirect effects on the success of individual fish [2,9,10]. Direct mortality can be decreased by a reduction in fishing effort, modifications to gear, or by avoiding times and places where there is high density of the species in question [11,12]. Avoidance of indirect effects on the resources needed by fish is most commonly achieved through spatial measures such as Marine Protected Areas (MPAs) or fisheries closures [13,14]. Understanding the role of juvenile commercial fish habitat can be particularly important since juvenile gadoid survival has been observed to have the greatest effect on population recruitment (e.g. [15–17]).

Protecting areas important to fish remains a less common approach to conserving fish than reducing fishing effort or modifying gear impact [2,18]. This is mainly because of the difficulties in understanding which habitat components are important to fish and how the extent (area of each substratum type) and heterogeneity (diversity and pattern of substratum types and patches within a landscape) of substrata affect fish populations [3,7,19]. Nonetheless, with the rise in spatial protection measures, knowledge of the distribution of species and their habitat is increasingly important [20,21].

Within coastal areas, mapping has been derived from aerial and satellite images. Use of optical imaging techniques in high visibility waters can provide useful information. Unfortunately such techniques are less useful in more turbid waters [3]. Acoustic methods can also provide detailed maps of the seabed [22], but can be resource intensive and prohibitively expensive [23,24]. Predictive methods can therefore be an important tool to overcome such issues [25]. Predictive outcomes will, however, vary according to the spatial scales used [26–28]. For individual MPAs and other spatial management plans to be effective, adequate spatial scales need to be used [26,28,29].

Given the need for higher resolution seabed maps to implement adequate spatial management measures, and the lack of knowledge of landscape effects on fish abundance and distribution, the aims of this study were two-fold. Firstly a range of environmental variables were used to undertake fine-scale predictive mapping of substrata within South Arran Nature Conservation MPA (NCMPA) [5]. The predicted seabed map was then used to understand how substratum type, extent and heterogeneity affected juvenile *G*. *morhua*, *M*. *aeglefinus* and *M*. *merlangus* relative abundance.

## Material and methods

### Study location

**Fig 1:**
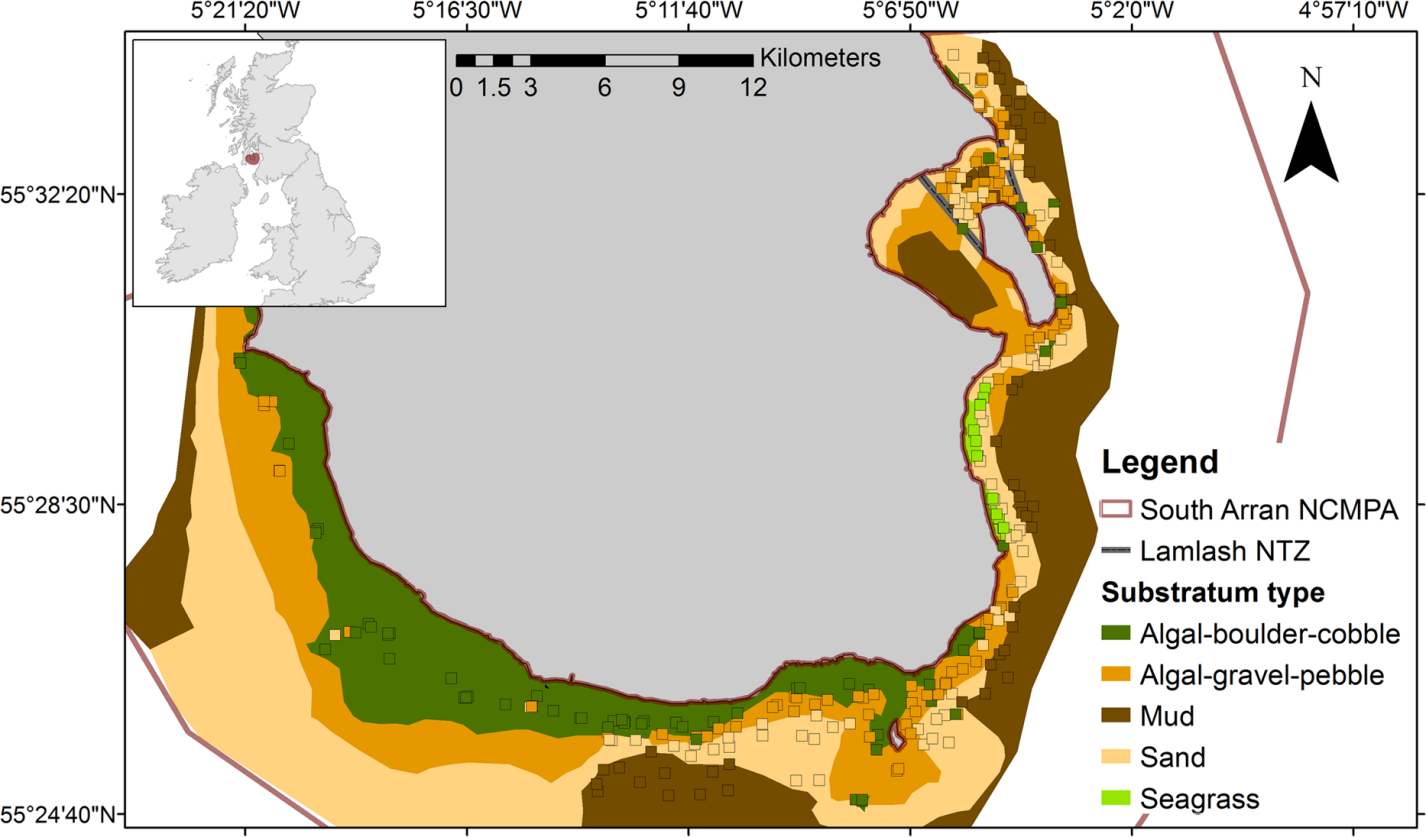
South Arran Nature Conservation Marine Protected Area, with substratum predictions in combination with ground-truthed data. Squares represent substratum types from stereo-video deployments.

Research was conducted from June to September 2013 and 2014 within the recently designated (2014) South Arran NCMPA, located within the Firth of Clyde, southwest coast of Scotland (Fig 1). The MPA encompasses an area of 250 km^2^ and contains within its boundaries a 2.67 km^2^ No Take Zone (NTZ) [30].

### Data collection

Gadoid sampling was undertaken using three Stereo Baited Remote Underwater Video (SBRUV) frames as described in [5]. The maximum number of individuals of each gadoid in the field of view at the same time (MaxN) [31,32] was used to assess the relative abundance of gadoids within each deployment. The methods used were based on those of [33,34], with modifications for lower light and visibility (< 6 m). Still images were extracted from the SBRUV and stereo-video SCUBA transect recordings to classify substrata and undertake substratum prediction modelling. The substratum types comprised of seagrass (*Zostera marina*), sand, mud, algal-gravel-pebble (AGP) which contained maerl, and algal-boulder-cobble (ABC). Two divisions of the Wentworth grain scale [35] were used to classify sediment type [36]. Refer to [5,37] for a detailed description of substratum categorisation. A total of 289 ground-truthed data points were used for substratum prediction analysis consisting of 258 SBRUV deployments and 31 SCUBA transects (Fig 1).

### Substratum model environmental descriptors

Five abiotic environmental predictor variables were used for predictive substratum modelling (Table 1). Depth data were obtained from vessel echosounders. Distance from shore was calculated using the GPS fix made at the time of deployment and ArcGIS v. 10.1 (EDINA digimap, British National Grid 1984). Maximum tidal speed was obtained from a three-dimensional Finite Volume Community Ocean Model (FVCOM) implementation for the Firth of Clyde [38,39]. The maximum tidal speed, evaluated over a whole year, was modelled at the location of each data collection point for the deeper layer of the model, as described by [38]. Wave fetch values were used as described in [40]. Information on the underlying geology of the MPA was obtained from the British Geological Survey (BGS) and downloaded from EDINA.

**Table 1:** Summary of environmental predictors.

Predictor	Description	Unit	Range
Depth	Water depth	m	4.0–47
Wave fetch	A measure of exposure of a shore (the distance which wind-driven waves can build from the closest land point)	km	193–2877
Distance to nearest coast	Distance of SBRUV from the shore	m	10–2295
Maximum tidal speed	Maximum tidal speed at spring tides in the deeper layer	ms^-1^	0.1–0.9
Geology	Dominant rock type found to occur in the area	Categorical	2 levels: Permian rock and Triassic rock

### Substratum predictive modelling

A multinomial generalized linear model was applied in order to undertake substratum distribution modelling. A multinomial distribution was selected since five possible discrete outcomes restricted between zero and one were plausible [41]. nnet R package in R software (v. 3.03, R Core Team, 2015) was used for the multinomial model. Prior to statistical analysis, continuous variables were standardised by dividing the mean by the standard deviation. One SBRUV deployment was removed as a result of an erroneous depth reading. Automated model selection was undertaken to find the model of best fit using the difference between Akaike’s Information Criterion (AIC) scores. Eq 1, provides the model of best fit for substratum prediction.

$$Y_{i}=\beta_{1},{Distance}_{i}+\beta_{2},{Depth}_{i}+\beta_{3},\;Max\;{Current}_{i}+\beta_{4},{Fetch}_{i}+\beta_{5},{Geology}_{ij}+\beta_{6},{Distance}_{i}*\beta_{7},{Depth}_{i}+\beta_{8},{Fetch}_{i}*\beta_{9},{Distance}_{i}$$ (1)

Where *Y*_*i*_ is the response variable and β are the modelled coefficients for sample *i*.

The multinomial model performance was tested on 25% of the collected SCUBA and SBRUV substratum dataset, by randomly splitting the data into 217 samples (75%) to fit the data and 72 samples (25%) to validate the data. To evaluate the accuracy of the model of best fit, an area under the curve (AUC) was performed [42] to give the percentage of correct predictions. Correct classification of the individual substratum categories was calculated to understand sensitivity of the model’s predicted substratum types. ROCR R package was used to discern how well each of the variables explained the presence of the modelled substrata.

To undertake substratum prediction modelling for the wider area, the environmental variables were extracted at each node point of the hydrodynamic model [38]. Depth data for the wider area was sourced from the General Bathymetric Chart of the Oceans (GEBCO) overlapped with SeaZone (v. 1.1). The predictor variables were then standardised, and the multinomial model of best fit was used to predict substratum type. The resulting data frame containing substratum predictions was imported into ArcGIS, converted into a point shapefile and validated with ground-truthed substratum types. Polygons were created joining the ground-truthed and predicted substratum data points to create a smooth continuous surface for each substratum type across the predicted area (Fig 1).

### Gadoid landscape calculations

Very little information exists on *in situ* juvenile gadoid movement patterns due to the difficulties in using acoustic tags on such small fish (below 10 cm) [43] and difficulties in tracking marked, recaptured individuals [44–46]. The horizontal distance and time which the juveniles moved within the field of view of the cameras was measured to calculate the speed and approximate distance they can move within one hour. To undertake these distance calculations, the cruising speeds (when the gadoids were moving in a straight line away from the bait) of ten individuals of each gadoid species of less than 20 cm were used.

Existing literature on gadoid swimming speeds and home ranges was also gathered (i.e. [44,46–49]). Both measures (distance speed calculations and information from existing literature) were used since, fish behaviour around baited cameras is not usually classed as normal, whereas existing literature on juvenile gadoid movement is sparse and varied. From the combined distance speed calculations and existing literature on gadoid speed and movement behaviour, a radius of 1500 m (covering an area of 7.07 km^2^) was created in ArcGIS around the gadoid point data collection.

Hill number N_∞_ (inverse of Berger-Parker dominance index) [50,51], was used to understand how landscape heterogeneity affected gadoid relative abundance, using the predicted substratum point data. N_∞_ gives the reciprocal of the proportional abundance from the most common specie [51,52], or in this case substratum type. Substratum richness was therefore the number of types of substrata within the 1500 m radius, and evenness the frequencies of the substrata within the radius. Hill numbers are inclusive of well-known indices and are expressed as the effective numbers [53,54]. N_∞_ (substratum dominance) was used since N_1_ (exponential of Shannon) and N_2_ (the inverse of Simpson’s index) are more sensitive to varying sample size [55,56]. The extents of each substratum polygon within the radii were calculated in ArcGIS.

A negative binomial error distribution using R package glmmADMD was used to model SBRUV fish counts. Random effects to account for varying location and grouped days of data collection were incorporated into the models where significant. Year was included into the model due to slight differences in sampling between years. Stepwise backwards selection was used for model selection by AIC minimisation. Tukey tests were performed to test for differences between substratum categories using the R package multcomp [57].

## Results

### Substratum distribution model

Evaluation of the multinomial model using the validation dataset indicated ‘excellent’ predictive power [41] (AUC score of 0.88) (l = -131.21, d.f. = 32, *p* < 0.001). Correct classification for seagrass was 100%, followed by mud with 89%. AGP, sand and ABC accurate classification received scores of 70%, 69% and 68% respectively. Predictor variable individual AUC scores were particularly strong for wave fetch and depth (0.71 and 0.69 respectively). Distance from coast and maximum tidal speed had AUC scores of 0.66 and 0.62.

### Landscape effects on gadoids

**Fig 2:**
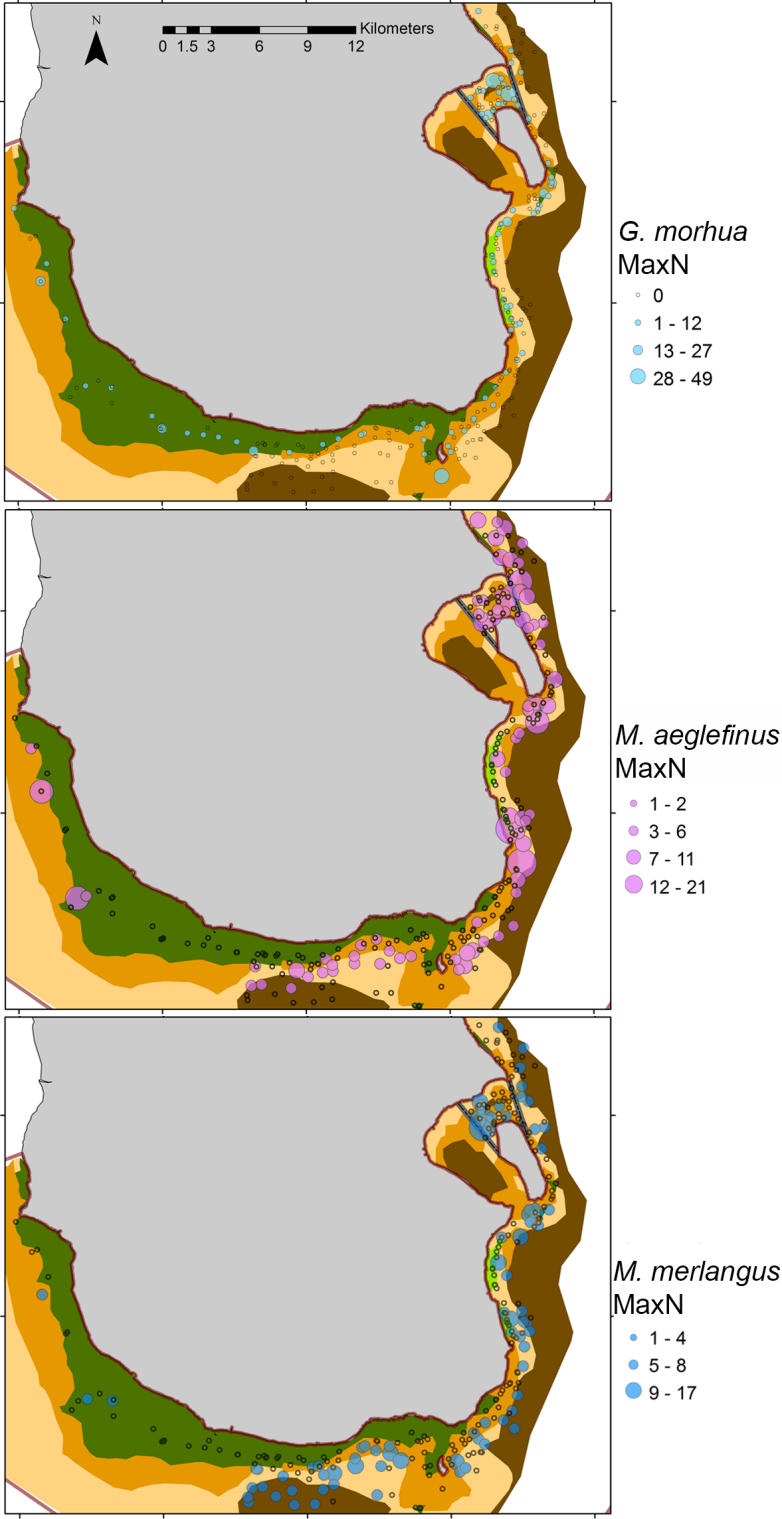
Substratum map with relative abundance bubble plots for juvenile *Gadus morhua*, *Melanogrammus aeglefinus* and *Merlangius merlangus*.

*G*. *morhua* highest MaxN was observed over AGP substratum type with the lowest average MaxN observed over sand (Table 2), no *G*. *morhua* were observed over mud. A decrease in MaxN was observed with increasing substratum dominance (N_∞_) (l = -311.87, d.f. = 9, θ = 0.98, *p* < 0.001; Table 3; Fig 2; Table A in S1 File).

**Table 2:** Juvenile gadoid MaxN substratum association summary results.

Substratum type	*Gadus Morhua*		*Melanogrammus aeglefinus*		*Merlangius merlangus*	
	MaxN	Mean MaxN ± s.e.	MaxN	Mean MaxN ± s.e.	MaxN	Mean MaxN ± s.e.
Algal-boulder-cobble	38	0.84 ± 0.22	3	0.07 ± 0.26	4	0.09 ± 0.29
Algal-gravel-pebble	338	4.57 ± 0.51	33	0.45 ± 0.23	32	0.43 ± 0.19
Mud	0	0	39	0.87 ± 0.19	53	1.20 ± 0.17
Sand	22	0.67 ± 0.30	158	2.03 ± 0.24	130	1.67 ± 0.26
Seagrass	52	1.38 ± 0.48	21	1.31 ± 1.15	3	0.19 ± 0.31

**Table 3:** Details from statistical models describing juvenile gadoid response to landscape variables. Arrows indicative whether the predictor variable significantly increased or decreased gadoid MaxN. N_∞_ refers to the dominance of the most common substratum type.

Species	Predictor variable	Significant predictor variable effect on gadoid MaxN	Substratum Tukey test significance
***Gadus morhua***	Substrata	ABC< AGP	*p* < 0.01
	N_∞_	↓	
	Extent	None	
***Melanogrammus aeglefinus***	Substrata	ABC < AGP	*p* < 0.05
		ABC < Mud	*p* < 0.001
		ABC < Sand	*p* < 0.001
		ABC < Seagrass	*p* < 0.05
	N_∞_	↑	
	Extent	None	
***Merlangius merlangus***	Substrata	ABC < AGP	*p* < 0.01
		ABC < Mud	*p* < 0.001
		ABC < Sand	*p* < 0.001
	N_∞_	↑	
	Extent	↑	
	N_∞_: Extent	↑	

*M*. *aeglefinus* and *M*. *merlangus* highest MaxN was observed over sand, with lowest MaxN observed over ABC (Table 2). An increase in *M*. *aeglefinus* and *M*. *merlangus* MaxN was observed with increasing substratum dominance (N_∞_) (l = -286.45, d.f. = 9, θ = 1.36, *p* < 0.001; Table 3; Fig 2; Table B in S1 File). A slight increase in *M*. *merlangus* MaxN was also observed with increasing substratum extent (l = -272.45, d.f. = 11, θ = 0.88, *p* < 0.001; Fig 2; Table C in S1 File).

## Discussion

This research demonstrates the importance of considering landscape effects on demersal fish for conservation and fisheries spatial management purposes. Using a range of predictor variables the distribution and configuration of seabed was able to be predicted within an MPA, saving on resource intensive costs of acoustic monitoring methods. The substratum prediction model enabled the heterogeneity and the extent of different substratum types, at ranges of up to 1500 m around gadoid data collection locations to be linked to gadoid relative abundance.

The multinomial model used for substratum prediction performed well. Depth and wave fetch had the greatest influence on substratum prediction. Depth affects many species and is commonly used as a surrogate for light, temperature and benthic shear stress from ocean swell [23,58]. Wave fetch and maximum tidal speed will also have an effect on benthic shear stress and light attenuation through varying levels of exposure and turbidity [23,24,59]. Maximum tidal speed additionally provided point-specific information of hydrodynamic regimes within the area.

Individual substratum correct classification showed that seagrass and mud substrata had the highest classification accuracy. Seagrass only grows in shallow areas where sufficient light can penetrate [24], and mud was only observed within deeper or more sheltered areas. ABC substrata also occurred at shallower depths (< 20 m) and more exposed areas composed of larger sediment grain sizes, where the macro-algae can anchor itself [58–60]. AGP substratum type largely consisted of varying percentages of maerl (coralline red algae, *Phymatolithon calcareum*), which together with red algae requires a lesser degree of light penetration (≤ 30 m) [61]. Sand can be found within a broad range of environmental conditions which may have led to sand having a lower prediction accuracy [62].

Using the predicted full coverage map, seabed landscape effects on gadoid relative abundance were modelled at a range relevant to the movement behaviour of the gadoids studied. *In situ* landscape effects on commercially important gadoid fish have not previously been explored [8]. Research on landscape effects of marine organisms has demonstrated the importance of such larger scale processes on the distribution of fish. For example [8] explored a range of landscape metrics on demersal reef fish relative abundance within a 200 m radius of SBRUV deployments in south-eastern Australia. A combination of different landscape measures, including distance from the reef and the length of the edges of the reef were found to influence their distribution.

*G*. *morhua* were observed over a variety of substrata occurring at shallower depths, with higher relative abundance observed over AGP containing maerl. *G*. *morhua* have a checkerboard brown and white colour which lend them to being more difficult to distinguish in gravel-pebble substratum types [37,63]. This substratum may therefore be favoured as an anti-predator mechanism. *G*. *morhua* are also known to inhabit shallow coastal waters where algal-gravel-pebble and algal-boulder-cobble occur [5,37,64]. Conversely *M*. *aeglefinus* and *M*. *merlangus* were observed in higher relative abundance over sand and mud. *M*. *aeglefinus* and *M*. *merlangus* have been known to occur in deeper waters than *G*. *morhua*, where sand and mud are more prevalent within the Firth of Clyde [5]. The species specific segregation observed between these gadoids may result in reduced interspecific predation and competition [65,66].

A decrease in *G*. *morhua* relative abundance was observed with increasing N_∞_ (substratum dominance). Indicating that juvenile *G*. *morhua* are associated with more heterogeneous landscapes rather than landscapes dominated by one specific substratum type. *M*. *aeglefinus* and *M*. *merlangus* were observed in higher relative abundance with increasing substratum dominance. *M*. *merlangus* were also observed in higher relative abundance with increasing substratum extent. The increase in *G*. *morhua* observed with increasing landscape heterogeneity may enable *G*. *morhua* to access areas with possibly increased food availability and areas with sufficient refuge [8,67,68]. An experimental study undertaken by [69] demonstrated that juvenile *G*. *morhua* seem to differentiate between substratum types, selecting areas where growth and survival were highest. The increase in *M*. *aeglefinus* and *M*. *merlangus* observed with increasing substratum dominance, and extent for *M*. *merlangus*, may be an indication of how these gadoids are better adapted to prey found within sand and mud substrata.

Few demersal species are associated with a single seabed type, but instead use a combination of substrata according to foraging, shelter and tidal behaviours [68,70]. Substratum boundaries are also thought to be important foraging and refuge areas for fish depending on the extent of the patches [69,70]. Attempts to identify fish nursery areas are frequently static processes identifying individual homogeneous seabed types [68]. However, seascapes are often dynamic and varied and the focal species may also undergo ontogenetic shifts as a result of changing resource needs [5,68,71]. Such behaviour (foraging, shelter and ontogenetic shifts in resource), may have explained why all three gadoids were observed over the range of substratum types and *G*. *morhua* were observed in higher relative abundance in more heterogeneous landscapes.

## Conclusion

This study demonstrates the importance of considering landscape measures in demersal fish distribution given that landscape heterogeneity had differing effects on the gadoids studied here. This is in stark contrast with the typical approaches of either disregarding habitat requirements, or seeking to ensure the availability of a single seabed type. The use of a range of environmental variables enabled an accurate prediction of substrata and the creation of a fine scale seabed map. The approaches used in this study could be applied on a larger scale for the selection of areas for stock improvement or trialled on other species. For *G*. *morhua*, the selection and prioritisation of more heterogeneous landscapes for protection as nurseries should be prioritised.

## Supporting information

S1 File *Gadus morhua*, *Melanogrammus aeglefinus* and *Merlangius merlangus* models of best fit. (DOCX)
