# Supplementary material for: Landscape effects on demersal fish revealed by field observations and predictive seabed modelling
Source: PLoS One. 2017 Dec 11;12(12):e0189011. doi: 10.1371/journal.pone.0189011 (PMC5724865; doi:10.1371/journal.pone.0189011)
Supplement: S1 File — (DOCX) [file pone.0189011.s001.docx]

**S1 File. *Gadus morhua,* *Melanogrammus aeglefinus* and *Merlangius merlangus* models of best fit**

Output from the model of best fit for the response variable A) *Gadus morhua* B) *Melanogrammus aeglefinus* C) *Merlangius merlangus* MaxN. Explanatory variables include substratum type, landscape heterogeneity (N_∞_), extent and Year. Coefficients and diagnostics (*z*-and *p*-values) indicate the effect of each parameter level on the reference level, denoted as intercept. The reference level is substratum type algal-boulder-cobble (ABC) and year 1 (2013).

**Table A. *Gadus morhua* model of best fit**

|  | **Estimate** | **Standard error** | ***z* value** | ***p*-value** |
| --- | --- | --- | --- | --- |
| **Intercept** | 1.83 | 0.85 | 2.16 | <0.03 |
| **Algal-gravel-pebble** | 1.41 | 0.44 | 3.25 | <0.00 |
| **Sand** | -0.32 | 0.51 | -0.61 | 0.54 |
| **Seagrass** | -0.18 | 0.75 | -0.23 | 0.81 |
| **N∞** | -0.76 | 0.34 | -2.22 | <0.03 |
| **Year 2** | -1.28 | 0.63 | -2.02 | <0.04 |

**Table B. *Melanogrammus* *aeglefinus* model of best fit**

|  | **Estimate** | **Standard error** | ***z* value** | ***p*-value** |
| --- | --- | --- | --- | --- |
| **(Intercept)** | -3.15 | 0.94 | -3.36 | <0.00 |
| **Algal-gravel-pebble** | 2.12 | 0.70 | 3.03 | <0.00 |
| **Mud** | 3.63 | 0.78 | 4.68 | <0.00 |
| **Sand** | 4.19 | 0.73 | 5.78 | <0.00 |
| **Seagrass** | 2.58 | 0.83 | 3.10 | <0.00 |
| **N∞** | 0.25 | 0.29 | 0.87 | 0.39 |
| **Year 2** | -1.13 | 0.69 | -1.64 | 0.10 |

**Table C. *Merlangius merlangus* model of best fit**

|  | **Estimate** | **Standard error** | ***z* value** | ***p*-value** |
| --- | --- | --- | --- | --- |
| **(Intercept)** | -8.21 | 2.34 | -3.51 | <0.00 |
| **Algal-gravel-pebble** | 3.82 | 1.17 | 3.26 | <0.00 |
| **Mud** | 5.10 | 1.29 | 3.97 | <0.00 |
| **Sand** | 5.32 | 1.22 | 4.38 | <0.00 |
| **Seagrass** | 3.31 | 1.41 | 2.35 | <0.02 |
| **N∞** | 1.35 | 0.66 | 2.03 | <0.04 |
| **Extent** | 0.00 | 0.00 | 2.71 | <0.01 |
| **N∞:Extent** | 0.00 | 0.00 | -2.17 | <0.03 |
| **Year 2** | -0.30 | 0.56 | -0.53 | 0.60 |
